# Examining the linkages between maternity services and postpartum modern contraceptive adoption among young women in India: Insights from the 2015–16 and 2019–21 National Family Health Survey

**DOI:** 10.1371/journal.pone.0289701

**Published:** 2023-08-09

**Authors:** Monirujjaman Biswas, Anuradha Banerjee

**Affiliations:** Centre for the Study of Regional Development, Jawaharlal Nehru University, New Delhi, Delhi, India; University of Salamanca, SPAIN

## Abstract

**Background:**

The adoption of maternity services and postpartum modern contraception are the two most crucial components that help in reducing maternal and infant mortality; still, India is consistently struggling with it. This paper, therefore, aimed to examine the linkages between use of maternity services and postpartum modern contraceptive adoption.

**Data and methods:**

The required reproductive calendar data were extracted from the 2015–16 and 2019–21 National Family Health Survey (NFHS) datasets. The assessment was made based on a sample of currently married women aged 15–24 years who had given most recent childbirth in five years preceding the survey. For the analysis, a time-to-event approach was applied using the Kaplan-Meier survival statistic, Log-Rank Chi-square test and Cox-Proportional Hazard (Cox-PH) models.

**Results:**

The results revealed that the proportion of postpartum modern contraceptive uptake among young users increased by 9%, from 33% in 2015–16 to 42% in 2019–21. The Cox-PH models revealed that, in both NFHS waves, the associations between various components of maternity services and postpartum modern contraceptive uptake were strongly significant, even after controlling for selected socio-economic and demographic correlates.

**Conclusions:**

The findings of this study reinforced urgent need for implementing integrated maternal-child health and family planning programmes and for boosting effective family planning counselling by health professionals to promote and motivate young women with a desire to early adoption of modern contraception in subsequent months after a recent childbirth.

## Introduction

Postpartum family planning (PPFP) has long been considered as a crucial component in improving both maternal and child survival [1]. Contraceptive uptake during the postpartum period is the key for preventing high-risk unplanned and unintended pregnancies, enhancing optimal birth spacing, averting the widespread unmet need for family planning (FP), and associated adverse maternal and child health outcomes [1, 2–4]. Globally, over 90% of women want to either delay or avoid their future pregnancies during the postpartum period [5]. An early exposure to pregnancy within 24 months following a live birth is associated with an increased risk of maternal and infant mortality [6, 7]. To reduce these rates, the World Health Organization (WHO) recommended that birth spacing between a woman’s previous birth and her latest pregnancy should be at least 24 months [8, 9].

It is estimated that nearly 2,72,000 maternal deaths worldwide and 86,366 maternal deaths in India occur annually, which could have been appreciably precluded by postpartum contraceptive uptake [10]. Recently, the 2019–21 National Family Health Survey (NFHS) reported that the Infant Mortality Rate (IMR) had dropped from 41 to 35 per 1000 live birth [11]. Although many efforts have been made by the Government to reduce the Maternal Mortality Rate (MMR) and Infant Mortality Rate (IMR), India is still lagging behind what it should achieve, according to the 2017 National Health Policy (NHP) targets [12]. Postpartum women are generally regarded as the most motivated to avoid the risk of having another child and, thus, to initiate contraceptive uptake immediately after a childbirth. The adoption of postpartum modern contraception remains low in many low and middle-income countries [13]. In India, nearly 42% of young married women were using postpartum modern contraception by the end of the first year during 2019–21 [11].

Most of maternal deaths could be prevented if women receive proper health care during and following pregnancy and childbirth [4]. Consequently, women are more likely to access medical health facilities for better reproductive health during maternal and post-delivery periods than at any other stage in life [14]. For instance, a study based in India highlighted that nearly 84% of women visited health centres to utilize maternal health care [15]. Notably, India has made significant progress towards utilizing various uptake of maternity services [Antenatal Care (ANC) visits, Place of Delivery (PoD) and Postnatal Care (PNC) check-up] in the past few decades. According to the latest 2015–16 and 2019–21 NFHS, young married women who received at least four or more ANC visits increased by 5% (from 53% to 58%), institutional delivery increased by 8% (from 84% to 92%) and PNC check-up within 42 days after delivery increased by 13% (from 70% to 83%), respectively [11, 16]. However, the utilization of maternity services during maternal and postpartum periods offers a crucial “gateway” to motivate users for early initiation of postpartum contraceptive uptake to avoid adverse pregnancy outcomes [15, 17]. Previous studies have also suggested that the utilization of maternity services can substantially enhance modern contraceptive uptake during the postpartum period [18–22].

It is recommended that FP counselling is a key for improving the health-care-seeking behaviour of women during the maternal and postpartum periods [8, 23, 24]. Existing literature has suggested that both the maternal and postpartum period offers a “window of opportunity” for women to interact with health workers during the utilization of maternity services with an intention to receive FP counselling advice that might enhance their future possibility of postpartum contraceptive uptake [18, 21]. In light of this, the Maternal and Child Health (MCH) centre would be considered as the best institution to motivate women for early initiation of modern contraceptive uptake in the immediate post-delivery period, and this motivation works as strong associations between the use of maternity services and postpartum modern contraceptive uptake. Furthermore, the extent of integration of maternity services with PPFP offers a range of potential benefits to improve postpartum modern contraceptive uptake. First, the integrated services are conceded as the most effective mechanism, which may reduce the time-related costs of accessing all services at the same location. Second, young women who receive integrated services may likely be exposed to PPFP counselling and promotional efforts by health professionals. Third, women who routinely receive maternity services have a range of invaluable opportunities to interact with health providers, which may motivate them in developing trust and satisfaction with the healthcare system. This would eventually repress misconceptions, myths and uncertainties and improve the broader cultural acceptability of postpartum modern contraception. Thus, these mechanisms may be relevant to address widespread unintended pregnancies and lengthen subsequent birth intervals [18].

In India, the MCH policies and programmes are recommended as the most viable strategy to improve PPFP services, which is under-emphasized by policymakers and stakeholders. Although the MCH programmes have been consistently updated and implemented overtime towards the realization of safe motherhood, the PPFP has integrated into maternity services through the National Population Policy (NPP, 2000), National Health Policy (NHP, 2002), the Reproductive and Child Health Programme (RCH, Phase I-1997–2004, Phase II-2005–10), the National Rural Health Mission (NRHM, 2005–12) and the Reproductive, Maternal, Newborn, Child Health and Adolescents (RMNCH+A, 2013) programmes [25–29]. To address the contraceptive needs of postpartum women, the Government has also implemented several initiatives under the National Family Welfare Program, including home contraceptive delivery by ASHA staff, Mission Parivar Vikas, awareness of the new and reversible contraceptive basket of choice, etc.

However, a surprisingly limited range of studies based on developed and developing countries, including India, have focused on linkages between the use of maternity services and postpartum contraceptive uptake. For instance, a study by Do and Hotchkiss (2013) observed strong associations between both combined and separate intensity of ANC and PNC with postpartum modern contraceptive uptake in Kenya and Zambia [18]. Using multi-country-based Demographic and Health Survey (DHS) datasets, a study by Zerai and Tsui (2001) reported that ANC has significantly influenced the early adoption of postpartum contraception even after controlling for other characteristics [30]. Unlike Zerai and Tsui (2010), Hotchkiss et al. (2005) showed a positive correlation between the intensity of maternity services and modern contraceptive uptake within a year of childbirth in Bolivia, Guatemala, Indonesia, Morocco, and Tanzania [17]. Several other studies have also demonstrated significant relationships between various uptake of maternity services and subsequent use of postpartum modern contraception [19, 31–38]. In the South Asian region, particularly in India, only a few research studies have explored the associations on this subject [15, 39, 40]. For example, a study by Dixit, Dwivedi and Gupta (2017) highlighted that the use of maternity services have emerged as significant factors of postpartum contraceptive adoption among married reproductive-aged women in 2005–06 [15]. More recently, another study by Bansal et al. (2022) that constructed an MCH index comprising factors on ANC, PNC and child vaccination suggested a significant association between MCH care and early initiation of contraception after delivery among married reproductive-aged women using 2015–16 NFHS data [39]. Hence, in light of the aforementioned literature and to address the knowledge gap in the existing research on this specific topic in the Indian context, the present study would feasibly be the first attempt to examine the linkages between the use of maternity services and postpartum modern contraceptive adoption amongst young married women using 2015–16 and 2019–21 NFHS reproductive calendar data. To shed light on this issue, the findings of this paper may generate significant interest among the MCH–FP programme planners and policymakers because if the use of maternity services does significantly influence postpartum modern contraceptive adoption, subsequently stepping into the evidence-based MCH–FP policies and programmes formulation would emerge as a feasible strategy to enhance contraceptive uptake among young postpartum women.

## Materials and methods

### Data source

The present study used the unit-level data from the latest two successive waves of the National Family Health Survey (NFHS) conducted in 2015–16 and 2019–21 [11, 16]. It is a cross-sectional survey carried out under the stewardship of the Ministry of Health and Family Welfare (MoHFW), Government of India (GoI). A stratified, multistage cluster sampling design was adopted to collect the nationally representative sample of households. The datasets are available in the public domain from the DHS data repository and can be accessed upon request (https://www.dhsprogram.com/data/dataset_admin); therefore, they did not require any ethical approval for conducting this specific study. Details of each survey’s sampling design, methodology, data processing and questionnaires can also be available in the respective survey report [11, 16]. Both NFHS waves provide reliable and up-to-date estimates of population, health and nutrition-related parameters as well as data on various socio-economic and programmatic dimensions at the district, state/Union Territory (UT), and national levels. In the history of NFHS, like 2015–16 NFHS, 2019–21 NFHS also provides district-level estimates on key demographic and health-related indicators, including fertility, mortality, FP, maternal and child health, immunization, anthropometric measurements, fertility preference, hypertension, blood glucose levels etc. [11, 16]. In 2015–16, NFHS was conducted in two phases (from 20 January 2015 to 4 December 2016) and interviewed a nationally representative sample of 699,686 eligible women aged 15–49 years and 103,525 eligible men aged 15–54 years from 601,509 households [16]. Similarly, in 2019–21, NFHS was conducted in two phases (from 17 June 2019 to 30 April 2021) and interviewed 724,115 eligible women aged 15–49 years and 101,839 eligible men aged 15–54 years from 636,699 sampled households [11].

For the study purpose, information was obtained from the reproductive calendar data included in the DHS-based Women’s Questionnaire, which was typically administered month-wise to cover all events of each woman’s experiences related to births, pregnancies, terminations, as well as contraceptive adoption for 60 months preceding the date of interview. Using contraceptive calendar data of NFHS, the postpartum modern contraceptive uptake was considered up to the first 12 months after a recent childbirth in five years prior to the survey month. The study was restricted to the first 12 months ‘post-pregnancy or postpartum period’ because both mother and child hold a higher risk of adverse outcomes during the specified period [36]. In addition, information on various components of maternity services (ANC, PoD and PNC check-up) were also extracted from the NFHS waves. To examine the linkages between the use of maternity services and postpartum modern contraceptive uptake, the sample of the study included only currently married women aged 15–24 years who had given most recent childbirth (excluding twins) in five years before the survey. As a result, the final study sample for the analysis was restricted to 38,750 (2015–16) and 34,389 (2019–21) young married women in India.

### Event of interest

The outcome of interest of this paper was the postpartum modern contraceptive uptake [pills, intrauterine devices, injectables, implants, condoms (male/female), diaphragm, foam/jelly, sterilization (male/female), standard days method, lactational amenorrhea method and emergency contraception]. It was constructed as a dichotomous variable with coded ‘1’ for young users who had adopted any modern contraception in the previous 12 months immediately after having the most recent childbirth, while those who had reported using any traditional contraception (rhythm, periodic abstinence or withdrawal and other traditional methods) or did not use any contraception were coded as ‘0’. For the outcome of interest, the duration (in months) was obtained from the date of most recent childbirth of a young woman to the time when she had started using modern contraception within the first 12 months postpartum or post-delivery period with the possibility of censoring. Censoring is a condition where the value of the unit of analysis is partially known. Thus, this time duration was considered to be appropriate for the survival analysis.

### Explanatory variables

The key independent variables of interest used in this paper were the utilization of maternity services using all three key programmatic indicators of maternity services relating to the most recent childbirth in five years preceding the survey, namely, number of ANC visits, PoD and PNC check-up. Dummy variables were created to indicate binary responses corresponding to each programmatic indicator. First, the number of ANC visits was coded as ‘1’ for young women who had attended 4 or more ANC visits, while less than 4 ANC visits were coded as ‘0’. Second, PoD was categorized as ‘1’ for young women who had given a most recent childbirth at a health facility (public/private); otherwise, those who delivered at a home setting were coded as ‘0’. Third, the PNC check-up was classified as ‘1’ for young mothers who received a PNC check-up within 42 days following a last birth and ‘0’ otherwise. In addition, a list of control variables were also included in the present analysis.

### Control variables

Based on the existing literature on this subject, a range of socio-economic and demographic variables were considered to be appropriate to control for in the multivariate analyses, examining the linkages between the use of maternity services and postpartum modern contraceptive adoption [15, 17–19, 23, 30, 35–41]. The variables included for the analysis were: place of residence (urban and rural); region of residence (north, central, east, north-east, west and south); women’s education (illiterate, primary, secondary and higher); religion (Hindu, Muslim and Others); caste [SC/ST (Scheduled Castes/Tribes), OBC (Other Backward Castes), and Others]; wealth index (poorest, poor, middle, richer and richest); mother’s age at last birth (15–19 and 20–24 years); parity (1, 2 and 3 or more); child loss (no and yes); desire for more children (wanted soon, wanted later and wanted no more); pregnancy intentions (wanted, mistimed and unwanted); distance to nearest health facility (no problem and minor & big problem) and media exposure to FP [no exposure, exposure through print media (newspaper/magazine/wall painting or hoarding) and exposure through electronic media (radio/television)]. Table 1 presents the summary statistics of the selected variables used in this study.

**Table 1:** Percentage distribution of young married women by selected background characteristics, India, NFHS, 2015–16 and 2019–21.

Variables	2015–16	2019–21
	*N* = 36,952 (%)	*N* = 32,259 (%)
**Place of residence**		
Urban	9345 (25.29)	7416 (22.99)
Rural	27607 (74.71)	24843 (77.01)
**Region of residence**		
North	4474 (12.11)	3687 (11.43)
Central	8171 (22.11)	7245 (22.91)
East	10758 (29.11)	10613 (32.9)
North-east	1196 (3.24)	926 (2.87)
West	5090 (13.77)	4152 (12.87)
South	7263 (19.65)	5439 (16.86)
**Women’s education**		
Illiterate	7205 (19.50)	4407 (13.66)
Primary	5396 (14.6)	3565 (11.05)
Secondary	21559 (58.34)	20946 (64.93)
Higher	2792 (7.56)	3339 (10.35)
**Religion**		
Hindu	30277 (81.94)	27059 (83.88)
Muslims	5123 (13.86)	4103 (12.72)
Others	1552 (4.20)	1068 (3.38)
**Caste**		
SC/ST	12812 (34.67)	11984 (37.15)
OBC	16380 (44.33)	14255 (44.19)
Others	7759 (21)	6016 (18.65)
**Wealth index**		
Poorest	7921 (21.44)	7849 (24.33)
Poor	8897 (24.08)	7784 (24.13)
Middle	8509 (23.03)	7161 (22.20)
Richer	7369 (19.94)	5971 (18.51)
Richest	4256 (11.52)	3494 (10.83)
**Mother’s age at last birth**		
15–19	7800 (21.11)	6278 (19.46)
20–24	29152 (78.89)	25981 (80.54)
**Parity**		
1	21774 (58.92)	19313 (59.87)
2	12317 (33.33)	10523 (32.62)
≥3	2861 (7.74)	2423 (7.51)
**Child loss**		
No	31885 (86.29)	27556 (85.42)
Yes	5067 (13.71)	4703 (14.58)
**Desire for more children**		
Want soon	34501 (93.37)	29978 (92.93)
Want later	1785 (4.83)	1807 (5.60)
Want no more	666 (1.80)	474 (1.47)
**Pregnancy intentions**		
Wanted	34291 (92.8)	29772 (92.29)
Mistimed	1949 (5.28)	1945 (6.03)
Unwanted	712 (1.93)	542 (1.68)
**Distance to nearest health facility**		
No problem	11783 (31.89)	12355 (38.30)
Minor & Big problem	25169 (68.11)	19904 (61.70)
**Media exposure to FP**		
No exposure	10966 (29.68)	8291 (25.70)
Exposure through print media	4301 (11.64)	3429 (10.63)
Exposure through electronic media	21685 (58.68)	20539 (63.67)

All ‘*N*’ and ‘%’ were based on weighted observations.

### Statistical analyses

Descriptive statistics were calculated to summarize the nature of the selected study variables and to describe the postpartum modern contraceptive adoption and various components of maternity services among young married women in India. The cumulative proportion of postpartum modern contraceptive uptake up to the first 12 months were also illustrated. In this paper, the event of interest was whether a young woman started using a modern contraception within 12 months following a most recent childbirth and after how many months she started to do so. For this, survival analysis was considered to be appropriate. Here, the ‘event’ (failure) was postpartum modern contraceptive uptake, and the ‘duration’ (time) was the number of months following a last childbirth at which a young user started adopting modern contraception, which was measured up to 12 months (follow-up period). Young married women (15–24 years) who had not started using contraception within 12 months postpartum period were considered survivors but not followed up. Hence it did not know if they had adopted modern contraception by the time of the survey. There were no left-censoring cases in the current study sample; young women who had given most recent childbirth (less than 12 months) and had not started using a postpartum modern contraceptive in five years before the month of the survey were considered right-censored cases. Moreover, the Kaplan-Meier (K-M) survival statistic was used in the analysis to present survival probabilities, generating K-M curves for various categories of key explanatory variables and selected other potential correlates. The differentials in postpartum modern contraceptive use by use of maternity services and selected background characteristics of young women were further investigated for the purpose of selecting variables in the regression analysis at the subsequent stage, using the Log-Rank Chi-square test. Lastly, the Cox-Proportional Hazards (Cox-PH) model was employed to investigate how the factors were associated with a specific event (e.g., time-to-postpartum modern contraceptive adoption) at a particular point in time. It accounted for censored survival times and allowed individual characteristics to change over time. For this reason, a time-to-event approach was adopted to deal with young postpartum users irrespective of whether or not they had adopted modern contraception by the time of the study. However, the Cox-PH regression models were used to estimate hazard ratios, examining the unadjusted effect of each of the study variables and the adjusted effect of all three components of maternity services on postpartum modern contraceptive uptake after controlling for selected background characteristics. Associations were summarized by using Unadjusted Hazard Ratios (UHR) and Adjusted Hazard Ratios (AHR) with a 95% Confidence Interval (CI). Further, a pair-wise correlation matrix test was applied to detect the possible multicollinearity; none of the pairs showed a correlation greater than 0.5. With regard to the multi-stage design for sample selection, sampling weights were carried out in the analysis to ensure accurate representativeness in the probability of selection and to adjust for non-responses across the country, stratified by place and region of residence [11, 16]. For this reason, the ‘*svyset’* command was applied to account for the effect of the complex survey design followed by NFHS. All statistical analyses were performed using STATA version 14.0 [42].

## Results

### Characteristics of the respondents

Table 1 summarizes the sample distribution of the selected socio-economic and demographic characteristics of young married women in India. As shown in Table 1, we observed that nearly three-fourths of respondents (74.7% in 2015–16 and 77.0% in 2019–21) resided in their rural counterparts. With respect to the level of education, in both NFHS waves, the proportion of women who had received no formal education decreased by only 5.8%, from 19.5% to 13.7%, followed by primary education (3.6%, from 14.6% to 11%), whereas the proportion of women who had completed secondary education increased by nearly 7%, from 58.3% to 64.9% followed by higher education (2.8%, from 7.6% to 10.4%), respectively. In terms of social group, both OBC and SC/ST communities were the most dominant social groups during 2015–16–2019–21. Regarding the distribution of household wealth status, about 46% in 2015–16 and 48% in 2019–21 of respondents were in the lowest two wealth quintiles, while the remaining quintiles stayed almost stable between surveys. Notably, over three-fourths of women (79% in 2015–16 and 81% in 2019–21) were between the ages of 20–24 years. More than 85% of the respondents had no child loss in both surveys. Also, the proportion of young married women in religion, parity, desire for more children and pregnancy intentions remained stable in both successive NFHS waves; the majority of young women (82%) belonged to the Hindu religion; approximately 59% of respondents had first, and 32% had second parity children; nearly 93% of women were each reporting a desire for another child soon and had wanted pregnancy intentions. Besides, the proportion of respondents who reported the distance to a nearest health facility for minor and big problems decreased by 6%, from 68% in 2015–16 to 62% in 2019–21. Lastly, more than half of the respondents had FP exposure through electronic media during 2015–16–2019–21.

### Use of maternity services and postpartum modern contraceptive uptake

**Fig 1:**
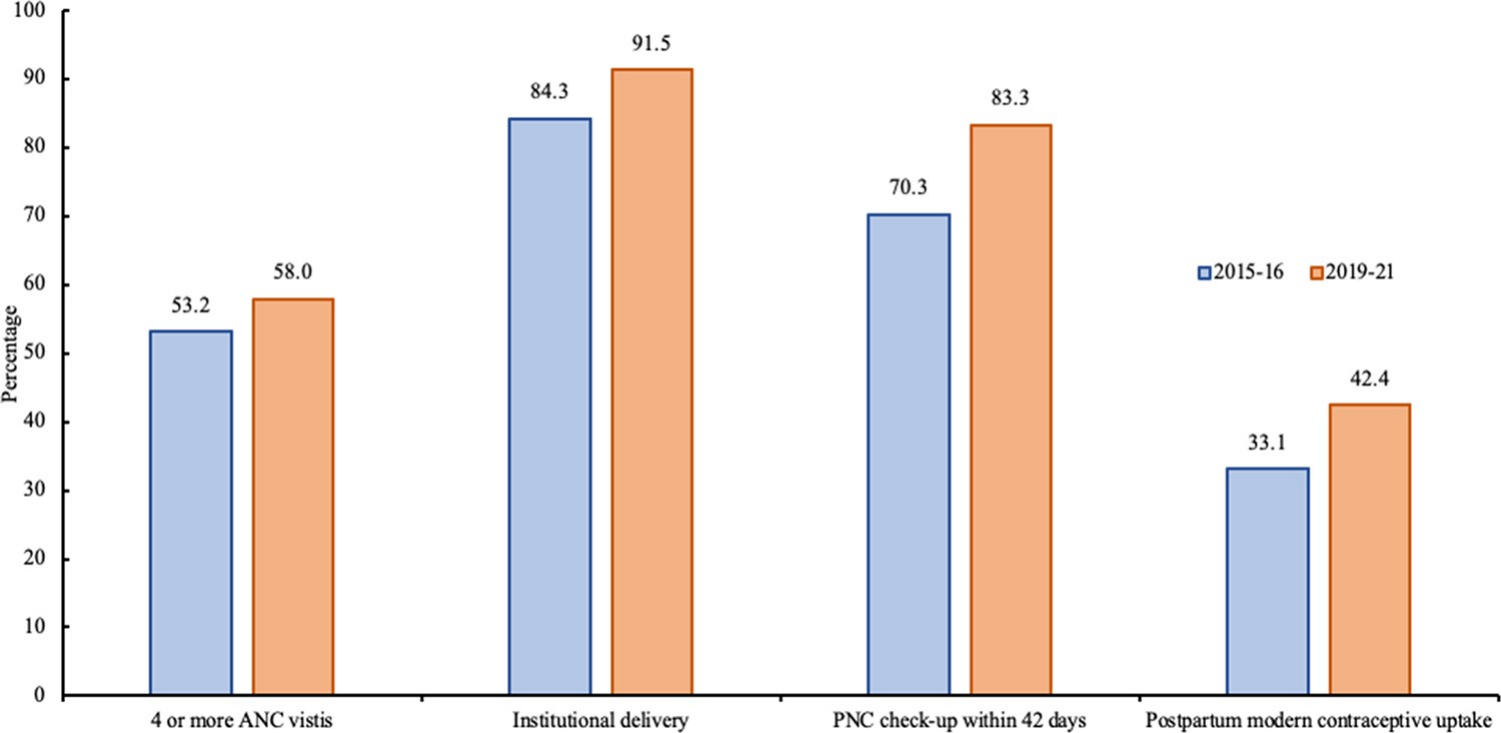
Percentage distribution of use of various components of maternity services and postpartum modern contraceptive uptake among young married women who had given a most recent childbirth, India, NFHS, 2015–16 and 2019–21.

Fig 1 shows the proportion of use of all three components of maternity services among young married women who had delivered a most recent childbirth. About 53% and 58% (increased by only 5%) of young women had attended 4 or more ANC visits during 2015–16–2019–21. Between 2015–16 and 2019–21, the proportion of young married women who had given a recent birth in an institutional setting increased considerably from nearly 84% to 92% (by 8%). Notably, the PNC check-up among mothers within 42 days after their most recent delivery had increased by 13%, from 70.3% in 2015–16 to 83.3% in 2019–21. Furthermore, Fig 1 illustrates the proportion of postpartum modern contraceptive adoption within the first 12 months following a latest childbirth among young married users had sharply climbed by only 9% point, from 33% to 42% during 2015–16–2019–21.

## Results from survival analysis

Based on the reproductive calendar data, Table 2 depicts a life table showing how young women started initiating postpartum modern contraceptive uptake following their most recent childbirth during 2015–16 and 2019–21, respectively. The table reported that the survivor probabilities of modern contraceptive uptake declined over the months, hinting at an upward trend (low uptake) in early initiation of modern contraception during the 12 months postpartum period.

**Table 2:** Survivor function of postpartum modern contraceptive uptake among young married women who had delivered most recent childbirth in five years preceding the survey, India, NFHS, 2015–16 and 2019–21.

Months	*N* (exposure)	Failure	Lost	Survivor function*	Standard error	95% CI
**2015–16**						
**1**	36889	2412	329	0.935	0.001	[0.932, 0.937]
**2**	34148	1163	468	0.903	0.002	[0.900, 0.906]
**3**	32517	1346	503	0.865	0.002	[0.862, 0.869]
**4**	30668	1208	462	0.831	0.002	[0.827, 0.835]
**5**	28998	906	346	0.805	0.002	[0.801, 0.809]
**6**	27746	944	330	0.778	0.002	[0.774, 0.782]
**7**	26472	901	315	0.752	0.002	[0.747, 0.756]
**8**	25256	539	165	0.735	0.002	[0.731, 0.740]
**9**	24552	431	129	0.723	0.002	[0.718, 0.727]
**10**	23992	395	112	0.711	0.002	[0.706, 0.715]
**11**	23485	327	101	0.701	0.002	[0.696, 0.706]
**12**	23057	313	81	0.691	0.003	[0.686, 0.696]
**2019–21**						
**1**	32190	4118	698	0.872	0.002	[0.868, 0.876]
**2**	27374	1534	704	0.823	0.002	[0.819, 0.827]
**3**	25136	1537	825	0.773	0.002	[0.768, 0.777]
**4**	22774	1342	795	0.727	0.003	[0.722, 0.732]
**5**	20637	975	548	0.693	0.003	[0.688, 0.698]
**6**	19114	1059	487	0.655	0.003	[0.649, 0.660]
**7**	17568	1060	495	0.615	0.003	[0.610, 0.621]
**8**	16013	506	276	0.596	0.003	[0.590, 0.601]
**9**	15231	432	191	0.579	0.003	[0.573, 0.584]
**10**	14608	296	155	0.567	0.003	[0.561, 0.573]
**11**	14157	272	116	0.556	0.003	[0.550, 0.562]
**12**	13769	294	122	0.544	0.003	[0.538, 0.550]

*: This showed the proportion of younger women were not using postpartum modern contraception till the corresponding month of follow up; CI: Confidence Interval.

**Fig 2:**
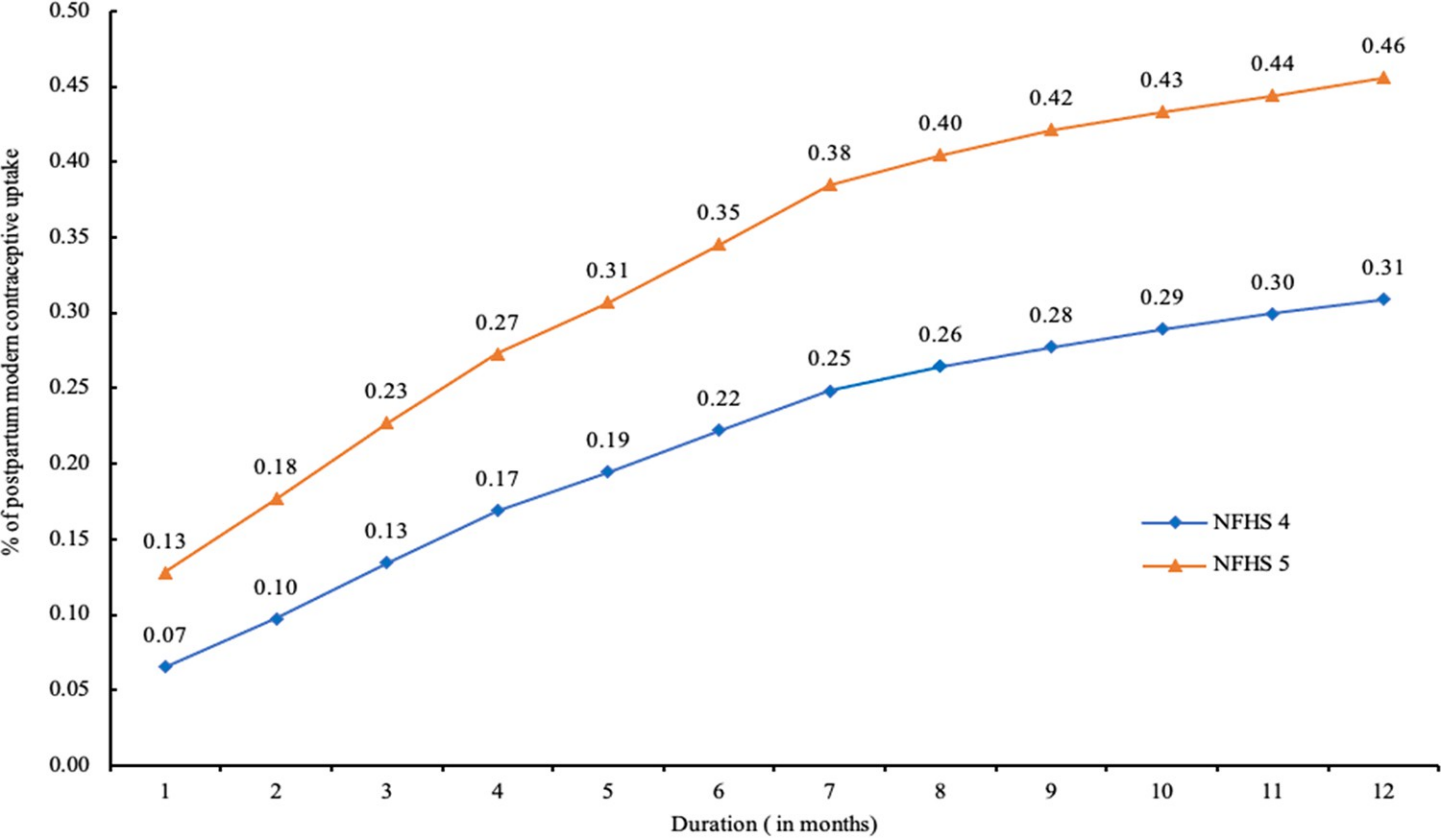
Cumulative proportion of young users who started using postpartum modern contraception by months from their most recent childbirth, India, NFHS, 2015–16 and 2019–21.

Fig 2 further displays the cumulative proportion of young users who had started using modern contraception within the first 12 months of the last childbirth. In 2015–16, only 7% of young users started using modern contraception by the end of the first month following a recent live birth. Subsequently, the proportion of young users had consistently used postpartum modern contraception over the months, adopting 13% by three months, 22% by six months, 28% by nine months, and 31% by the end of twelve months, respectively.

**Fig 3:**
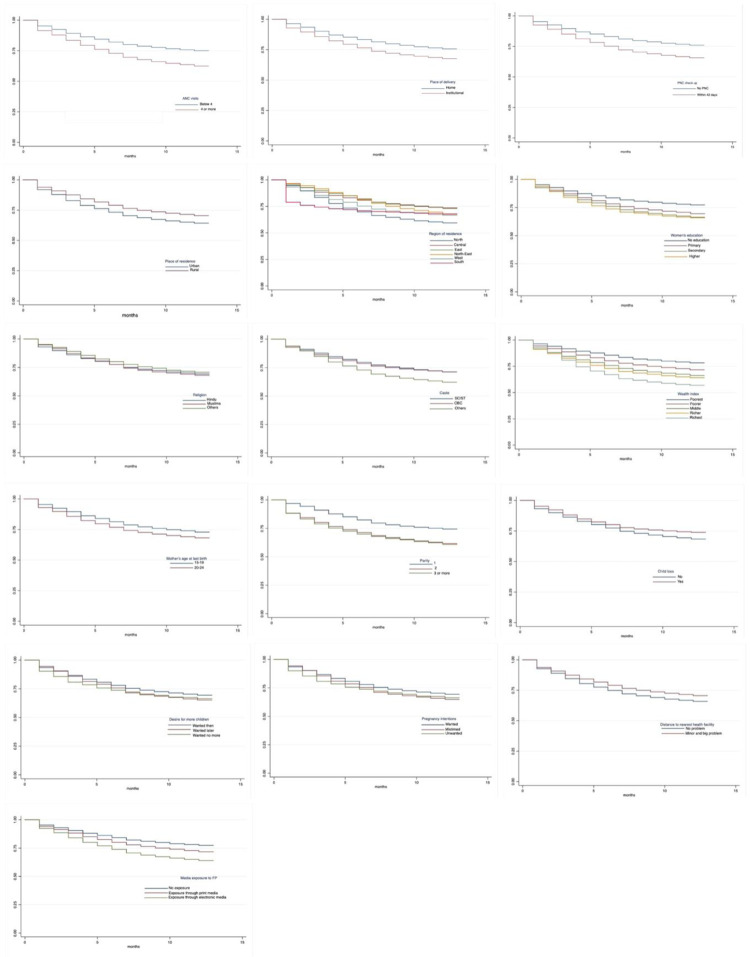
Kaplan-Meier survival curves of postpartum modern contraceptive uptake within 12 months following a latest childbirth by selected background characteristics, India, NFHS, 2015–16. (a) ANC visits. (b) Place of delivery. (c) PNC check-up. (d) Place of residence. (e) Region of residence. (f) Level of education. (g) Religion. (h) Caste. (i) Wealth index. (j) Mother’s age at last birth. (k) Parity. (l) Child loss. (m) Desire for additional child. (n) Pregnancy intention. (o) Distance to nearest health facility. (p) Media exposure to FP.

**Fig 4:**
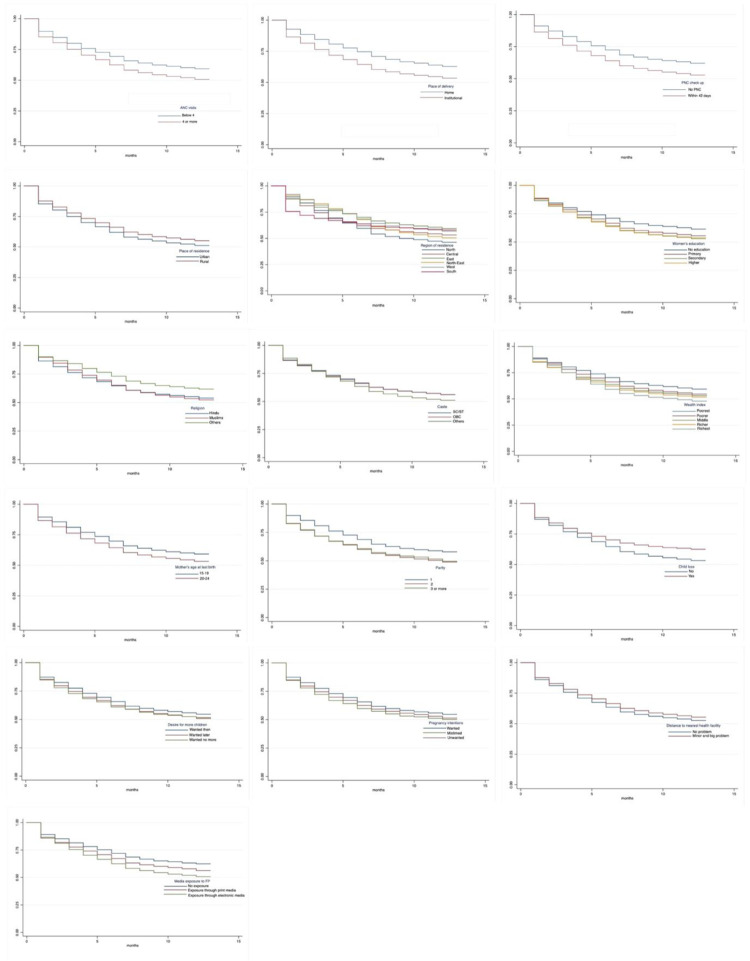
Kaplan-Meier survival curves of postpartum modern contraceptive uptake within 12 months following a last childbirth by selected background characteristics, India, NFHS, 2019–21. (a) ANC visits. (b) Place of delivery. (c) PNC check-up. (d) Place of residence. (e) Region of residence. (f) Level of education. (g) Religion. (h) Caste. (i) Wealth index. (j) Mother’s age at last birth. (k) Parity. (l) Child loss. (m) Desire for additional child. (n) Pregnancy intention. (o) Distance to nearest health facility. (p) Media exposure to FP.

The graphical illustrations in Figs 3 and 4 provide insights into key features of the differences in K-M survival curves of modern contraceptive uptake within 12 months following a last childbirth by use of various maternity services and selected other participants’ characteristics. The figures highlighted that the estimated K-M curves varied for young users who had reported the number of ANC visits, followed by PoD and PNC check-up during 2015–16–2019–21, respectively. Moreover, the K-M survival curves differed substantially for various categories of the respondent’s socio-economic and demographic characteristics such as place and region of residence, educational level, religion, caste, wealth status, mother’s age at last birth, parity, child loss, desire for more children, pregnancy intentions, distance to nearest health facility and media exposure to FP between 2015–16 and 2019–21. It is also evident, for instance, in 2015–16, the significant differences between the proportions of survivors were observed by place of residence, caste and parity, hinting very less proportion of postpartum modern contraceptive adoption by young women residing in urban location, affiliated with OBC and first parity children than those in rural counterparts, belonged to SC/ST or other castes and had higher parity children. Likewise, in 2019–21, the significant variances between the proportions of survivors were evidenced by religion, parity and child loss. Before we fit the Cox-PH models, the observed differences in K-M survival curves for various categories of the selected study variables were further assessed using the Log-rank test.

### Differentials in postpartum modern contraceptive uptake

Table 3 summarizes the results of the Log-rank test, examining significant differences in survival curves for the selected components of maternity services and other background characteristics of respondents (place of residence, region of residence, women’s education, religion, caste, wealth index, mother’s age at last birth, parity, child loss, desire for more children, pregnancy intentions, distance to nearest health facility and media exposure to FP). Barring religion (2015–16) and desire for additional child (2019–21), the results suggest that the survival functions for the adoption of postpartum modern contraception varied significantly for various categories of selected socio-economic and demographic correlates. Although the Log Rank test determined if there were any significant survival variances between the two groups, it did not assure how much difference they were actually. To address this gap, the Cox-PH hazards models were applied in the analysis.

**Table 3:** Results of Log-rank test (non- parametric) for equality of survivor functions of postpartum modern contraceptive uptake by selected background characteristics, India, NFHS, 2015–16 and 2019–21.

Characteristics	2015–16		2019–21	
	Chi square test (χ^2^)	*p*-value	Chi square test (χ^2^)	*p*-value
**ANC visits**	656.2	***0*.*0000***	219.9	0.0000
**PoD**	168.4	0.0000	115.7	0.0000
**PNC check-up**	387.1	0.0000	151.8	0.0000
**Place of residence**	122.3	0.0000	31.4	0.0000
**Region of residence**	453.2	0.0000	174.8	0.0000
**Level of education**	300.2	0.0000	93.1	0.0000
**Religion**	7.5	0.0241	68.3	0.0000
**Caste**	179.1	0.0000	23.2	0.0000
**Wealth index**	767.6	0.0000	154.3	0.0000
**Mother’s age at last birth**	67.8	0.0000	74.9	0.0000
**Parity**	793.2	0.0000	297.6	0.0000
**Child loss**	51.2	0.0000	90.6	0.0000
**Desire for additional child**	15.3	0.0005	12.4	0.0021
**Pregnancy intentions**	19.6	0.0000	13.8	0.0000
**Distance to nearest health facility**	82.1	0.0000	26.5	0.0000
**Media exposure to FP**	616.5	0.0000	279.7	0.0000

### Factors associated with postpartum modern contraceptive uptake

In the Cox-PH models, Tables 4 and 5 reports the results of the UHR and AHR of postpartum modern contraceptive uptake by key explanatory variables and selected other background characteristics of respondents. The unadjusted results denoted the contribution of each variable on the probabilities of using modern contraception during the specified postpartum period. Conversely, the adjusted results provided key insights into the changes in the impact of maternity services on the postpartum modern contraceptive uptake when the analysis was controlled for other background characteristics.

**Table 4:** Results obtained from the Cox Proportional Hazard model on postpartum modern contraceptive uptake within first 12 months following a most recent childbirth, by background characteristics, India, NFHS, 2015–16.

Characteristics	Bivariate		Multivariate	
	UHR	95% CI	AHR	95% CI
**ANC visits**				
≤4	1.000		1.000	
≥4	1.341***	[1.701, 2.593]	1.589***	[1.493, 1.691]
**PoD**				
Home	1.000		1.000	
Institutional	1.314***	[1.813, 2.395]	1.113***	[1.022, 1.212]
**PNC check-up**				
No PNC	1.000		1.000	
Within 42 days	1.304***	[2.161, 2.698]	1.274***	[1.186, 1.367]
**Place of residence**				
Urban	1.000		1.000	
Rural	0.808***	[0.754, 0.866]	0.964	[0.892, 1.041]
**Region of residence**				
North	1.000		1.000	
Central	0.603***	[0.563, 0.646]	0.755***	[0.702, 0.813]
East	0.897***	[0.835, 0.963]	1.049	[0.97, 1.135]
North-east	1.022	[0.944, 1.106]	1.124**	[1.02, 1.239]
West	0.947	[0.856, 1.047]	0.776***	[0.698, 0.862]
South	0.912**	[0.836, 0.996]	0.819***	[0.748, 0.896]
**Level of education**				
Illiterate	1.000		1.000	
Primary	1.553***	[1.404, 1.718]	1.331***	[1.203, 1.473]
Secondary	1.662***	[1.542, 1.790]	1.326***	[1.218, 1.443]
Higher	1.394***	[1.238, 1.571]	1.223***	[1.071, 1.396]
**Religion**				
Hindu	1.000		1.000	
Muslims	1.094**	[1.001, 1.196]	0.942	[0.85, 1.044]
Others	1.357***	[1.200, 1.535]	1.130**	[0.996, 1.283]
**Caste**				
SC/ST	1.000		1.000	
OBC	0.860***	[0.809, 0.914]	0.821***	[0.77, 0.875]
Others	1.315***	[1.215, 1.424]	1.133***	[1.042, 1.231]
**Wealth index**				
Poorest	1.000		1.000	
Poor	1.408***	[1.290, 1.537]	1.237***	[1.130, 1.355]
Middle	1.675***	[1.540, 1.822]	1.402***	[1.273, 1.544]
Richer	1.734***	[1.584, 1.899]	1.491***	[1.334, 1.666]
Richest	1.751***	[1.576, 1.946]	1.622***	[1.422, 1.850]
**Mother’s age at last birth**				
15–19	1.000		1.000	
20–24	1.128***	[1.055, 1.205]	0.915**	[0.851, 0.983]
**Parity**				
1	1.000		1.000	
2	1.882***	[1.782, 1.987]	2.250***	[2.121, 2.386]
≥3	1.888***	[1.726, 2.064]	2.733***	[2.461, 3.036]
**Child loss**				
No	1.000		1.000	
Yes	0.773***	[0.710, 0.841]	0.734***	[0.67, 0.804]
**Desire for additional child**				
Wanted soon	1.000		1.000	
Wanted later	1.182***	[1.056, 1.322]	1.175	[0.851, 1.624]
Wanted no more	1.295**	[1.049, 1.598]	0.903	[0.491, 1.659]
**Pregnancy intentions**				
Wanted	1.000		1.000	
Mistimed	1.191***	[1.071, 1.324]	0.857	[0.63, 1.165]
Unwanted	1.301***	[1.065, 1.589]	1.244	[0.699, 2.212]
**Distance to nearest health facility**				
No problem				
Minor & Big problem	0.879***	[0.831, 0.93]	0.964	[0.908, 1.024]
**Media exposure to FP**				
No exposure	1.000		1.000	
Exposure through print media	1.172***	[1.062, 1.294]	1.071*	[0.966, 1.189]
Exposure through electronic media	1.446***	[1.350, 1.549]	1.188***	[1.101, 1.280]

*p*-value: ***p < 0.001, **p < 0.01, *p < 0.05; UHR: Unadjusted Hazard Ratio; AHR: Adjusted Hazard Ratio; CI: Confidence Interval.

**Table 5:** Results obtained from the Cox Proportional Hazard model on postpartum modern contraceptive uptake within first 12 months following a most recent childbirth, by background characteristics, India, NFHS, 2019–21.

Characteristics	Bivariate		Multivariate	
	UHR	95% CI	AHR	95% CI
**ANC visits**				
≤4	1.000		1.000	
≥4	1.506***	[1.158, 1.958]	1.333***	[1.267, 1.403]
**PoD**				
Home	1.000		1.000	
Institutional	1.650***	[1.354, 2.012]	1.217***	[1.111, 1.332]
**PNC check-up**				
No PNC	1.000		1.000	
Within 42 days	1.897***	[1.611, 2.233]	1.193***	[1.107, 1.285]
**Place of residence**				
Urban	1.000		1.000	
Rural	0.904***	[0.85, 0.961]	0.936*	[0.873, 1.003]
**Region of residence**				
North	1.000		1.000	
Central	0.857***	[0.804, 0.914]	0.937*	[0.875, 1.003]
East	0.796***	[0.746, 0.849]	0.888***	[0.826, 0.955]
North-east	1.007	[0.938, 1.080]	1.076***	[0.979, 1.182]
West	0.713***	[0.646, 0.787]	0.684***	[0.615, 0.760]
South	0.865***	[0.801, 0.934]	0.822***	[0.759, 0.890]
**Level of education**				
Illiterate	1.000		1.000	
Primary	1.324***	[1.208, 1.451]	1.197***	[1.092, 1.312]
Secondary	1.361***	[1.268, 1.461]	1.231***	[1.142, 1.327]
Higher	1.303***	[1.182, 1.435]	1.169***	[1.050, 1.302]
**Religion**				
Hindu	1.000		1.000	
Muslims	1.081**	[1.009, 1.158]	0.974	[0.898, 1.058]
Others	1.001	[0.891, 1.123]	0.951	[0.845, 1.069]
**Caste**				
SC/ST	1.000		1.000	
OBC	0.969**	[0.921, 1.020]	0.935*	[0.886, 0.986]
Others	1.081**	[1.006, 1.161]	1.034	[0.961, 1.112]
**Wealth index**				
Poorest	1.000		1.000	
Poor	1.145***	[1.072, 1.223]	1.054**	[0.986, 1.126]
Middle	1.214***	[1.134, 1.299]	1.111***	[1.032, 1.196]
Richer	1.185***	[1.102, 1.274]	1.060***	[0.974, 1.154]
Richest	1.303***	[1.200, 1.415]	1.149**	[1.032, 1.278]
**Mother’s age at last birth**				
15–19	1.000		1.000	
20–24	1.180***	[1.112, 1.252]	1.049**	[0.982, 1.121]
**Parity**				
1	1.000		1.000	
2	1.422***	[1.358, 1.488]	1.565***	[1.489, 1.644]
≥3	1.398***	[1.288, 1.517]	1.749***	[1.597, 1.915]
**Child loss**				
No	1.000		1.000	
Yes	0.791***	[0.738, 0.848]	0.776***	[0.723, 0.833]
**Desire for additional child**				
Wanted soon	1.000		1.000	
Wanted later	1.112***	[1.012, 1.223]	1.318	[0.925, 1.879]
Wanted no more	1.137	[0.951, 1.359]	0.951	[0.623, 1.451]
**Pregnancy intentions**				
Wanted	1.000		1.000	
Mistimed	1.098***	[1.001, 1.206]	0.790	[0.557, 1.119]
Unwanted	1.161	[0.986, 1.368]	1.109	[0.754, 1.632]
**Distance to nearest health facility**				
No problem				
Minor & Big problem	0.924***	[0.882, 0.969]	0.955*	[0.910, 1.003]
**Media exposure to FP**				
No exposure	1.000		1.000	
Exposure through print media	1.23***	[1.127, 1.342]	1.170***	[1.072, 1.277]
Exposure through electronic media	1.39***	[1.314, 1.472]	1.299***	[1.224, 1.379]

*p*-value: ***p < 0.001, **p < 0.01, *p < 0.05; UHR: Unadjusted Hazard Ratio; AHR: Adjusted Hazard Ratio; CI: Confidence Interval.

As results were shown in Table 4, the use of maternity services were significantly and positively associated with postpartum modern contraceptive adoption even after controlling for other background characteristics, suggesting that its strength remained tha same and highly valuable in explaining the differentials in the likelihood of adoption of postpartum modern contraception in both bivariate and multivariate models. In other words, young women who had 4 or more ANC visits, institutional delivery and PNC check-up within 42 days after the delivery had a significantly higher likelihood of modern contraceptive uptake than those who received less than 4 ANC visits, home delivery and no PNC check-up after a childbirth. In contrast, the adjusted results showed that compared with less than 4 ANC visits, birth at home and no PNC check-up, the risk of starting the adoption of postpartum modern contraception was significantly higher for young users who had 4 or more ANC visits (AHR: 1.589, 95% CI: 1.493–1.691, *p*<0.001), institutional delivery (AHR: 1.113, 95% CI: 1.022–1.212, *p*<0.001) and PNC check-up within 42 days after the delivery (AHR: 1.274, 95% CI: 1.186–1.367, *p*<0.001). Thus, the results of both UHR and AHR suggested that the strength of associations between the use of various maternity services on postpartum modern contraceptive uptake was significantly reduced by other correlates. Furthermore, in bivariate models, respondents resided in urban areas, stayed in the northern region, attended primary, secondary and higher education, affiliated with Muslim and other religions, belonged to the top four wealth quintiles, mothers aged 20–24 years at last childbirth, higher parity of children, no child loss, desired to have another child later or no more, had intended, mistimed and unwanted pregnancy, no problem with distance to a nearest health facility and FP exposure through print and electronic media were strongly associated with a higher relative risk of using postpartum modern contraception within first 12 months of the postpartum period following a recent live birth.

However, even after adjusting for other correlates adopted in the analysis, a significantly higher likelihood of postpartum contraceptive uptake was observed among young women residing in the north-eastern region (AHR: 1.124, 95% CI: 1.020–1.239, *p*<0.01), had primary (AHR: 1.331, 95% CI: 1.203–1.473, *p*<0.001), secondary (AHR: 1.326, 95% CI: 1.218–1.443, *p*<0.001) and higher (AHR: 1.223, 95% CI: 1.071–1.396, *p*<0.001) education, affiliated with other religions (AHR: 1.130, 95% CI: 0.996–1.283, *p*<0.01) and castes (AHR: 1.133, 95% CI: 1.042–1.231, *p*<0.01), belonged to poor (AHR: 1.237, 95% CI: 1.130–1.355, *p*<0.001), middle (AHR: 1.402, 95% CI: 1.273–1.544, *p*<0.001), rich (AHR: 1.491, 95% CI: 1.334–1.666, *p*<0.001) and richest (AHR: 1.622, 95% CI: 1.422–1.850, *p*<0.001) wealth quintiles; users in second (AHR: 2.250, 95% CI: 2.121–2.386, *p*<0.001) and higher parity (AHR: 2.733, 95% CI: 2.461–3.036, *p*<0.001), had FP exposure through print (AHR: 1.071, 95% CI: 0.966–1.189, *p*<0.05) and electronic (AHR: 1.188, 95% CI: 1.101–1.28, *p*<0.001) media compared with those who had resided in the northern region, illiterate, Hindu, SC/ST, poorest quintile, first parity and no media exposure to FP. Unlike bivariate models, factors, namely, place of residence, desire for additional child, pregnancy intentions and distance to a nearest health facility, did not show any significant effect on the postpartum modern contraceptive uptake compared with the results of the multivariate analysis.

Table 5 also demonstrates that the adoption of various components of maternity services remained as the key significant factors of postpartum modern contraceptive uptake even after controlling for selected other background characteristics. The adjusted results further revealed that the relative risk of starting the adoption of modern contraception during the postpartum period was significantly higher for users who had 4 or more ANC visits (AHR: 1.333, 95% CI: 1.267–1.403, *p*<0.001), institutional delivery (AHR: 1.217, 95% CI: 1.111–1.332, *p*<0.001) and PNC check-up within 42 days after the delivery (AHR: 1.139, 95% CI: 1.107–1.285, *p*<0.001) than those who visited ANC for less than 4 times, birth at home setting and had no PNC check-up. Comparing both UHR and AHR, the results further highlighted that the relationships between use of maternity services and postpartum modern contraceptive adoption were significantly and relatively reduced by other factors.

Like Table 4, the bivariate models in Table 5 reported similar findings using 2019–21 NFHS data. After controlling for other potential correlates, a significantly higher likelihood of postpartum modern contraceptive uptake was observed among young users residing in the north-eastern region (AHR: 1.076, 95% CI: 0.979–1.182, *p*<0.01), had primary (AHR: 1.197, 95% CI: 1.092–1.312, *p*<0.001), secondary (AHR: 1.231, 95% CI: 1.142–1.327, *p*<0.001) and higher (AHR: 1.169, 95% CI: 1.050–1.302, *p*<0.001) education, belonged to the poor (AHR: 1.054, 95% CI: 0.986–1.126, *p*<0.01), middle (AHR: 1.111, 95% CI: 1.032–1.196, *p*<0.001), richer (AHR: 1.060, 95% CI: 0.974–1.154, *p*<0.01) and richest (AHR: 1.149, 95% CI: 1.032–1.278, *p*<0.01) wealth quintiles, mother’s aged 20–24 years at last birth (AHR: 1.049, 95% CI: 0.982–1.121, *p*<0.01), had second (AHR: 1.565, 95% CI: 1.489–1.644, *p*<0.01) and higher parity children (AHR: 1.749, 95% CI: 1.597–1.915, *p*<0.001), and FP exposure through print (AHR: 1.170, 95% CI: 1.072–1.277, *p*<0.01) and electronic (AHR: 1.299, 95% CI: 1.224–1.379, *p*<0.01) media than those who were from the northern region, had no formal education, belonged to poorest quintile, aged 15–19 years at last birth, first parity and no FP exposure. Unlike bivariate results, no significant impact on postpartum modern contraceptive uptake was observed in the multivariate analysis, especially for religion, mother’s age at last birth, desire for additional child and pregnancy intentions. Moreover, respondents from the rural counterparts, resided in the central, eastern, western and southern regions, belonged to OBC, had child loss, and distance to a nearest health facility for minor and big problems emerged as significant factors associated with adjusted lower risk of modern contraceptive uptake during the postpartum period.

The Cox-PH results reported that, in both Tables 4 and 5, the use of maternity services were strongly associated with postpartum modern contraceptive uptake even after controlling for other potential correlates. With regard to background characteristics, the results further highlighted the contrasting findings between 2015–16 and 2019–21 NFHS. Unlike Table 5 (bivariate models), the results in Table 4 revealed that respondents affiliated with other religions, desired no additional child and unwanted pregnancy intentions were significantly associated with a higher relative risk of postpartum modern contraceptive uptake. Comparing Table 5, the multivariate results revealed that users affiliated with other religions and castes were strongly associated with modern contraceptive uptake (Table 4). Interestingly, the relationship between mother’s aged 20–24 years at last birth and postpartum modern contraceptive uptake was significant, as shown in Table 5. It is worth mentioning that, in both unadjusted and adjusted results in Table 4, the adoption of postpartum modern contraception increased significantly with an increase in wealth status.

## Discussion

Based on the 2015–16 and 2019–21 NFHS reproductive calendar data, our results highlighted that despite the considerable improvement in all maternity indicators and postpartum modern contraceptive uptake, the early initiation of postpartum modern contraception was not up to the mark amongst young married women in India. And this finding could be of crucial importance for India’s FP programmes targeted at improving contraceptive uptake, especially during the critical postpartum period. Interestingly, the key finding of this study showed strongly positive associations between the use of maternity services (ANC, PoD and PNC check-up) and postpartum modern contraceptive uptake, even after adjusting for selected background characteristics in both NFHS waves. And this finding is well acknowledged in the existing literature on this subject and is largely consistent with the findings reported by earlier research studies [15, 17, 19, 31, 37, 39, 43, 44]. The findings of this paper underlined the importance of implementing the quality of health care services and providing FP information and counselling during the ANC visits, which could be deemed an ideal mechanism to encourage postpartum modern contraceptive uptake among young women in India. The study further denoted effective programmatic implications that the PPFP should be integrated into the MCH services to promote postpartum modern contraceptive uptake continuously. With this intention, the Government should play a foremost role in making a reliable institution for providing and monitoring all integrated Maternal-Child Health–Family Planning (MCH-FP) services, ensuring that all women can access and receive the potential benefits from the same institution, which is cost-effective and efficient in low resource settings [45]. Moreover, the findings of this paper underscored the importance of including FP counselling by health professionals to come in contact with young postpartum women at the time of utilising any maternity services (from the beginning of ANC visits to PNC check-up) at the MCH centre, which is also considered a key mechanism to improve modern contraceptive adoption, especially during the extended postpartum period.

The study results further identified that in both NFHS waves, a range of socio-economic and demographic factors (place of residence, level of education, caste, wealth status, mothers age at last birth, parity, child loss, distance to nearest health facility and media exposure to FP) were also significantly associated with subsequent use of postpartum modern contraception. And our findings are in agreement to some extent with the findings of previous studies conducted in India [15, 24, 39] and elsewhere [18, 19, 23, 36, 46]. The findings of this paper further implied that both well-educated and wealthiest young women might have better abilities in expressing their fertility desires or might have afforded the cost of FP services and received proper health education and other health care services from the healthcare system. Besides, previous studies have documented inconsistent pieces of credible evidence on linkages between postpartum modern contraceptive uptake and its driving forces. For instance, a recent study based in Nepal suggested that, apart from wealth status and media exposure to FP, women’s occupation, husband’s presence and fertility preference are also strongly associated with modern contraceptive uptake within 12 months following a recent childbirth [37]. While the current findings manifested that the desire for additional children and pregnancy intentions did not reflect any significant variances in the adjusted relative risk of modern contraceptive uptake in the immediate postpartum period. And this finding is differed from those of Kafle, Dulal & Pandey (2017), where the respondent’s place of residence, level of education and child parity have not shown any striking differences in the adjusted risk of early adoption of postpartum modern contraception [37].

Nevertheless, the findings of this paper offered key insights into the implementation of various policies and programmes that may help to improve modern contraceptive uptake, particularly during the critical postpartum period. Although India has made a steady progress towards achieving the Sustainable Development Goals (SDGs) targets by 2030, the MCH programme has emerged as a feasible strategy to enhance the PPFP services. Hence, our study findings suggested an undeniable need for implementing effective FP counselling and promotional efforts by health professionals and Community Health Workers (CHWs) to promote and encourage modern contraceptive uptake among young users during each maternal health visit. Apart from this, the Government should effectively devise and implement the integrated MCH-FP programmes towards strengthening and scaling up various maternity services that might be the most efficient and effective mechanism to motivate young women to early adoption of various contraceptive methods in the immediate post-childbirth in India. At the same time, the Government should place an imperative strategy to focus on inescapable PNC check-up within 42 days after delivery that would help in increasing FP practices, especially among socio-economically disadvantaged young users. However, several potential strategies should be considered while drafting policies and programmes, so as to: improve the availability and accessibility of various FP methods; focus on strengthening the coverage and quality of integrated MCH-FP services; educate more number of younger women; enrich male participation; and build a strong capacity for health care-seeking behaviours through health education and increasing interaction with health professionals and other health system contacts. These are the most promising avenues that would undoubtedly go a long way in enhancing the early initiation of contraception after the delivery. The study further reinforced an urgent need to promote the MCH-FP programmes among young women who have faced difficulties in accessing and utilizing the benefits of the MCH-FP services, which may undoubtedly be regarded as the most effective mechanism to substantially enhance postpartum modern contraceptive adoption across India, especially in low-resource settings.

This paper used the latest 2015–16 and 2019–21 NFHS data and was thus subject to study limitations. Using the contraceptive calendar approach, data on postpartum modern contraceptive uptake was collected month-wise, based on the large-scale cross-sectional NFHS data, which had become the standard practice [47], and reporting the history of contraceptive adoption in calendar structure was found to be superior in other standard forms [48]. Since the variable of interest of this paper was determined by using NFHS data, the probability of recall lapses in the reproductive calendar history was not excluded. It is worth mentioning that the NFHS waves were not used for establishing the cause-and-effect relationships between variables and were thus subject to the recall bias that may be associated with the collected retrospective contraceptive calendar data. Besides, the statistical analysis of this paper did not control the effect of the availability of FP practices, as the 2015–16 and 2019–21 NFHS data did not provide any information regarding the same. Also, both NFHS waves were not completely captured and under-or over-represented all episodes of contraceptive uptake within the first 12 months of the postpartum period following a recent live birth [49]. It is highly likely that the reproductive calendar information on month-wise contraceptive adoption in five years preceding the month of interview was subject to significant recall bias, which may have affected the data quality. Future research should take into account a range of community- and facility-level factors that may influence the early initiation of modern contraceptive uptake, examining the associations between the use of maternity services and postpartum modern contraceptive adoption amongst young women in India.

## Conclusion

In India, the adoption of modern contraception within 12 months following a recent childbirth was lowest amongst young married women but increased significantly between 2015–16 and 2019–21. The results revealed that all three programmatic indicators of maternity services were strongly associated with postpartum modern contraceptive uptake, even after adjusting for other potential correlates. The findings of this paper suggested that maternity services remain a crucial window of opportunity to provide access to FP information and to improve FP practices, especially during the extended postpartum period. This paper also highlighted that several socio-economic and demographic factors were significantly associated with postpartum modern contraceptive uptake in both rounds. Despite the government’s efforts to provide free maternity and FP services among women, India has yet to achieve its fertility goals. This may be due to lower contraceptive adoption, widespread unintended and closely spaced pregnancies and higher unmet need for FP [16]. However, it is indispensable to strengthen and implement the integrated MCH-FP programmes for providing the quality of MCH-FP services among young women with an intention to enhance modern contraceptive adoption immediately after childbirth. In addition to get the potential benefits of the integrated MCH-FP services, the present study further reinforced an urgent need for designing and implementing community-level programmatic interventions towards outreaching the FP counselling and health providers initiatives in effectively escalating knowledge and motivation among young postpartum women. This may increase the value of early initiation of contraceptive uptake for spacing or limiting future pregnancies and prevent adverse pregnancy outcomes, thereby improving women’s reproductive health behaviour and that of their children.
